# Aspirin in Children; are There Not Two Sides of the Same Coin?

**DOI:** 10.1007/s00246-024-03676-6

**Published:** 2024-10-09

**Authors:** Dietmar Schranz

**Affiliations:** Department of Pediatric Cardiology, Goethe University Clinic Frankfurt, Theodor-Stern-Kai 7, 60596 Frankfurt, Germany

**Keywords:** Aspirin, Dosage, Neonates, Congenital heart disease

## Abstract

Dose-dependent in vitro effects of aspirin on platelet inhibition and predictors of non-responsiveness have led to the recommendation of significantly higher doses of aspirin (5 mg/kg/day) in newborns and infants. The results are inconsistent with the pharmacodynamic effects of clopidogrel in newborns, where approximately 30% (0.2 mg/kg/day) of the adult dose (75 mg/day) showed equally effective antiaggregative effects. Consequently, the optimal aspirin dosage remains to be determined. The administration to newborns with congenital heart defects needs to address treatment goals, while accounting for the intricate interactions between platelets and endothelium, as well as the unique aspects of surgical and interventional procedures.

Regan IE et al. [1] conducted a highly interesting prospective observational study aimed at (i) evaluating the effects of aspirin dose on platelet inhibition and (ii) determining the prevalence and clinical predictors of aspirin non-responsiveness by utilizing platelet mapping (TEGPM) with emphasis on the MAAA (maximum amplitude with arachidonic acid) and in children > 2 years with additional LTA (light transmission aggregometry) techniques.

One key finding was that the tested platelets of all patients responded to aspirin in a dose of 5 mg/kg/day, while children > 2 years responded already to a dose of 3 mg/kg/day. The authors concluded that current practice could result in reduced platelet inhibition due to underdosing. Based on these in vitro tests, they recommended that younger children require higher doses of aspirin (5 mg/kg/day) inverse proportion to age.

However, these consecutive recommendations are in clear contradiction to the results of the Picolo® (Platelet inhibition in Children on Clopidogrel) trial [2] and the detected pharmacodynamic effects of clopidogrel in newborns and infants [3]. Given the lower baseline ADP/thrombin receptor-activating peptide (TRAP) percent platelet aggregation (PA) in neonates and infants, the pediatric clopidogrel dose of 0.2 mg/kg/day was found to be effective. This clopidogrel dose, which is 30–50% compared to the adult dose, induces a similar PA inhibition in neonates and infants and is significantly lower than the simple weight-based extrapolation of adult dose of 75 mg [3].

Therefore, from an in vivo perspective, doesn’t the author’s aspirin recommendation remain empirical if the other side of the coin, especially the age-dependent hemostasis namely the endothelial function, is also sufficiently taken into account? There is a clear consensus that excessive platelet activity leads to thrombosis. Thus, the therapeutic goal of aspirin use is to block inhibit the production of TXA2 (ThromboxanA2) in non-nuclear platelets without affecting vascular PGI2 production. This approach aims to protect a still viable vessel from thrombus formation, resulting in the current anti-thrombotic, “low dose” aspirin treatment regimen, which clinically neither affects the endogenous blood pressure regulatory mechanism nor anticongestive or antihypertensive drug treatment [4].

Of course, this study clearly indicates that irreversible and complete platelet inhibition in vitro is dose-dependent and may be age-dependent. However, what implications do higher doses have, particularly in relation to the lower baseline ADP/TRAP percent PA in neonates and infants, but also in terms of the dose-dependent pharmacological effects of aspirin on vascular PGI2 production, including its role as a dose-dependent arterial duct constrictor?

The implication that all neonates/infants should receive aspirin for thrombosis prophylaxis at a potentially anti-inflammatory (COX1 + COX2-inhibiting) dosage of 5 mg/kg/day [4] raises several questions regarding differential therapy:

1. Should the “empirical” neonatal aspirin treatment of 1–2 mg/kg daily, which is based on holistic medical considerations, be entirely replaced by aspirin monotherapy of 5 mg/kg/day, even in specific clinical constellations [5], where we combine clopidogrel in a daily dosage of 0.2 mg/kg with aspirin (1–2 mg/kg/day) to achieve high anti-thrombotic effectiveness while minimizing vascular side effects?
2. Should aspirin (5 mg/kg/day) be administered regardless of whether a non-endothelialized surgical (PTFE) shunt requires protection from thrombus formation or if a viable arterial duct is to be stented for pulmonary/systemic blood flow supply?
3. Did the authors consider or exclude r the vasoconstrictive effects of aspirin, particularly its a ductal constricting effects, at a dosage of 5 mg/kg/day?

In answering these specific questions, it is crucial to clarify the basis with additional information supporting the arguments made.

## Data Availability

No datasets were generated or analysed during the current study.
